# Capacitive Based Micromachined Resonators for Low Level Mass Detection

**DOI:** 10.3390/mi12010013

**Published:** 2020-12-25

**Authors:** Muhammad Umair Nathani, Haleh Nazemi, Calvin Love, Yameema Babu Lopez, Siddharth Swaminathan, Arezoo Emadi

**Affiliations:** Department of Electrical and Computer Engineering, University of Windsor, Windsor, ON N9B 3P4, Canada; nazemih@uwindsor.ca (H.N.); lovec@uwindsor.ca (C.L.); babulop@uwindsor.ca (Y.B.L.); swaminas@uwindsor.ca (S.S.); arezoo.emadi@uwindsor.ca (A.E.)

**Keywords:** capacitive, clamped membrane, gas detection, gas sensor, gravimetric, microbridge, microcantilever, micromachined resonator, microelectromechanical systems, sensitivity

## Abstract

Advancements in microfabrication technologies and novel materials have led to new innovations in miniaturized gas sensors that can identify miniscule changes in a complex environment. Micromachined resonators with the capability to offer high sensitivity and selectivity in array integration make mass loading a potential mechanism for electronic nose applications. This paper investigates the mass sensing characteristics of progressive capacitive based micromachined resonators as potential candidates for volatile organic compound detection where also there is a need for miniaturized array configuration. In this paper, a detailed investigative review of the major three geometric designs of capacitive based micromachined resonators, namely, the microcantilever, the microbridge and the clamped membrane sensors is performed. Although many reviews are present in literature regarding mass sensors, however there is a gap in the literature regarding the common capacitive based micromachined mass sensors. This research gives a review on the foundation for capacitive based micromachined mass sensors while highlighting the potential capabilities of each geometric design to be developed further. Moreover, this paper also introduces the advancements based on the geometric designs of the capacitive based micromachined mass sensors. An in-depth analysis is done for each geometric design, to identify the critical design parameters, which affect the sensors’ performances. Furthermore, the theoretically achievable mass sensitivity for each capacitive based micromachined mass sensor is modeled and analyzed using finite element analysis with mass variation in the picogram range. Finally, a critical analysis is done on the sensor sensitivities and further discussed in detail wherein each design is compared to each other and its current advances. Additionally, an insight to the advantages and disadvantages associated with each simulated geometry and its different advances are given. The results of the investigative review and analysis indicate that the sensitivities of the capacitive based micromachined sensors are dependent not only on the material composition of the devices but also on the varying degrees of clamping between the sensor geometries. In essence, the paper provides future research the groundwork to choose proper candidate geometry for a capacitive based micromachined mass sensor, with its several advantages over other mass sensors, based on the needed application.

## 1. Introduction

Mass sensing is one of the candidate methods used for bio-organic, chemical, and inorganic gas detection. This technique has garnered much interest in areas such as biomedical, automotive, military, and atmospheric monitoring. This paper introduces capacitive based micromachined resonators as a competitor technology for mass detection. This paper is focused on capacitive based sensors due to several advantages over other sensors based on piezoelectricity, acoustic, piezoresistive, optical, and more. It has been observed that the sensors working on the principle of capacity showed higher electromechanical coupling. Such sensors also benefitted with a higher bandwidth, a higher quality factor and easy integration with electronics through CMOS due to advances in microfabrication for micromachined devices. Moreover, these sensors possess an edge over others due to their reversibility, low cost due to microfabrication and higher selectivity due to employment of arrays without reducing the quality factor. Furthermore, such devices are a suitable niche to be reviewed due to their adaptability to different geometries, ease of measurement, and their design potential for batch fabrication for high sensitivity and low limit of detection applications in complex environments.

In this paper, an investigative critical review is done on the common geometries, namely the microcantilever, the microbridge, the capacitive clamped membrane sensors, and its consequent implications as capacitive based micromachined mass sensors. This is due to the several advantages of capacitive based micromachined mass sensors over others and the lack of a structured and concise foundation in the literature giving a much-required overview of the common capacitive based micromachined mass sensors. Additionally, the paper is focused on capacitive based sensors due to their design potential for batch fabrication for high sensitivity and low limit of detection applications in complex environments. The performance of the sensors is evaluated by their sensitivity, selectivity, reversibility, quality factor (QF), limit of detection (LOD), response time, and recovery period. Their circuit integration capability, size, and power usage are also key parameters, which make these sensors viable and feasible. In this work, these sensors are classified into three categories in congruence to their mass detection mechanism, sensitivity, and physical properties. This is followed by the finite element analysis (FEA)-based simulations of each sensor categorically with its sensitivity profile. Since the discussed geometries are foundational geometries on which many different capacitive based mass sensors have been developed, therefore, many advancements based on each sensor geometry have also been introduced. This is backed with an in-depth analysis and explanation of the advantages, disadvantages and the possible solutions to the cons related to each advancement coupled with comparative tabulated sensitivities related to each simulated design and its concurrent advancements. Consequently, these sensors are analyzed and evaluated against each other and their respective advancements to provide a thorough understanding of the strengths and the drawbacks for each sensor geometry. Therefore, effective groundwork is provided in this paper towards choosing an appropriate geometry for a capacitive based micromachined mass sensors as per its needed application.

## 2. Finite Element Analysis Validation and Mesh Refinement Study

**Figure 1:**
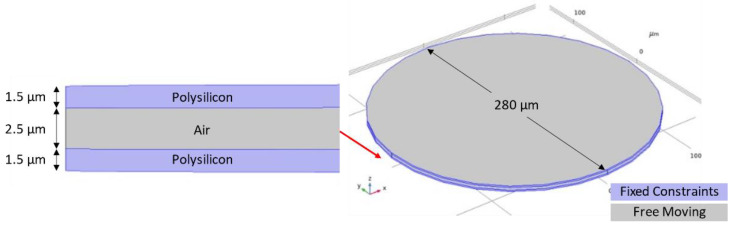
Clamped membrane structure used for validation and mesh refinement study.

The study done in this paper is validated from [1] wherein the resonant frequency obtained in the paper was 310 kHz whereas the simulation conducted resulted in a resonant frequency around 312 kHz with a deviation below 1%. The Multiphysics and the electrostatic and mechanical equations that were used in the finite element analysis simulations were the same for all structures. Therefore, this validation could be used for the microcantilever, microbridge and clamped membrane structures. Figure 1 shows the structure alongside its parameters and material properties that was used for validation.

**Figure 2:**
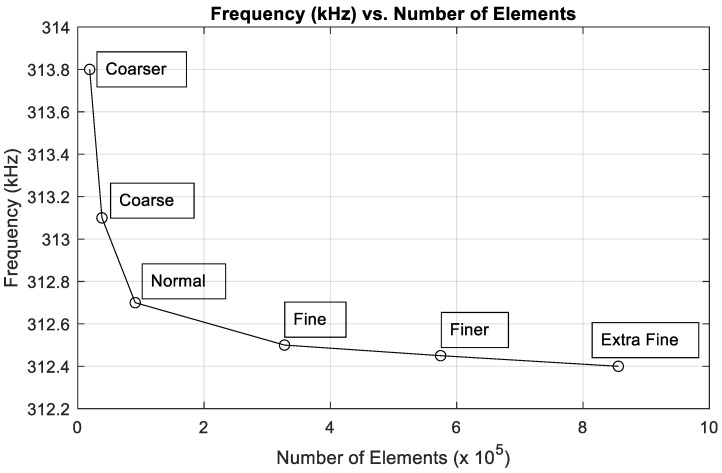
Mesh refinement study for microcantilever, microbridge, and clamped membrane structures.

Furthermore, a mesh refinement study is done in this paper for the simulated structure, the clamped membrane, as shown in Figure 1. This study is also applicable to all the following structures in the current scope of study due to the Multiphysics and the equations applied in the simulation being the same for all structures. Meshing is verified by varying the user-controlled meshing sequence for different mesh types such as COMSOL Multiphysics^®^ software’s Coarser mesh to extra fine mesh, as shown in Figure 2. The absence of any significant change in the resonant frequency while changing the mesh type indicates that the model’s result is independent of the number of meshing elements. The user-controlled fine mesh provides a result for which there was 0.02% of deviation observed on increasing the number of meshing elements. This ensures that the model achieves the same result while utilizing lesser computational resources and a faster simulation time. Thus, the fine mesh was chosen for simulating the devices in this paper.

## 3. Microcantilever Mass Sensors

### 3.1. Mechanism of Operation

**Figure 3:**
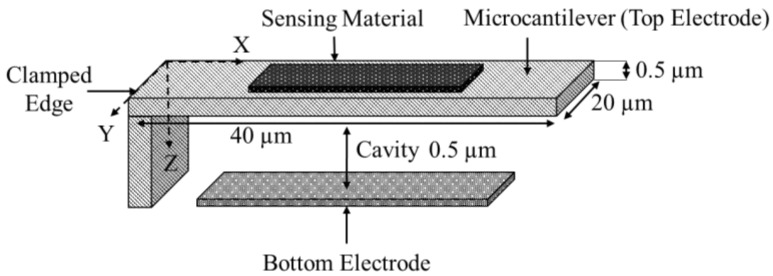
Schematic view of a microcantilever structure (image not to scale).

**Figure 4:**
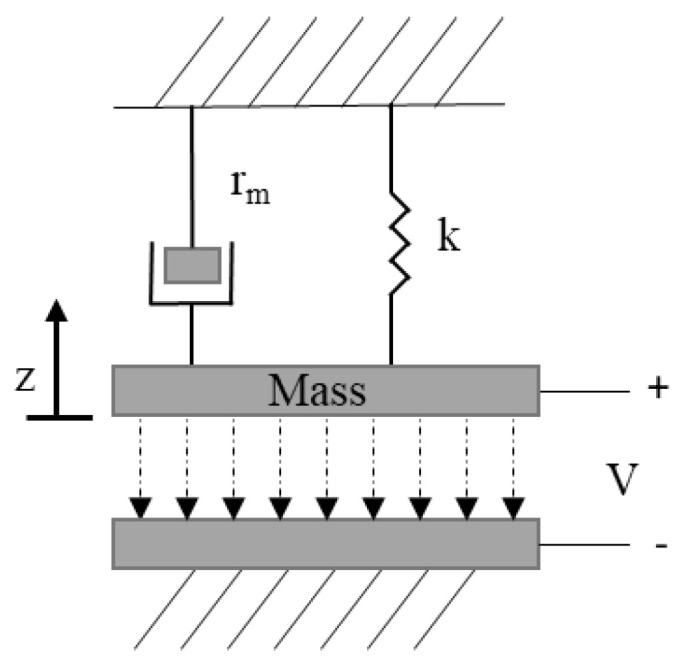
Mechanical model for microcantilever, microbridge, and capacitive clamped membrane.

Microcantilever mass sensors consist of a rectangular shaped beam, normally composed of silicon [2], which is clamped at one end to the substrate forming a flexible electrode. The unclamped end of the beam is suspended above the substrate where a second electrode is deposited [3], by a fixed and designed distance, as shown in Figure 3. Microcantilever mass sensor dimensions are typically up to 50 µm, 500 µm, and 2 µm for width, length, and thickness, respectively. However, dimensions ranging in the hundreds of nanometers have been achieved with emerging nanotechnology fabrication techniques [4]. The two electrodes are biased creating an electrostatic force that causes a deflection in the flexible electrode. The resonant frequency of the device is influenced by its physical and material properties and its biasing condition. This resonator is partially or fully functionalized by a sensing layer, deposited at the free end of the microcantilever structure to promote adsorption of the analyte of interest. This alters the mass of the suspended beam causing it to deflect towards the bottom electrode thereby shifting the resonant frequency of the device. By observing the shift in the resonant frequency of the device, the mass of the adsorbed analyte is determined [2,5,6,7,8]. The microcantilever can be modeled as a parallel plate capacitor represented by a mass spring damper model [9]. The damper, $r_{m}$, and the mass are used to model the medium acoustic impedance. The mechanical model that relates the electrostatic force to the counter-balancing mechanical forces influencing the behaviour of the device is shown in Figure 4.

In these sensors, the deflection amplitude of the flexible end of the microcantilever sensor is deduced by Hooke’s law, shown in Equation (1) [10,11,12],

(1) F=−kz

where F, k, and z represent the restoring force, the spring constant, and the out of plane displacement, respectively.

The proportional spring constant k is influenced by the dimensions and material composition of the flexible beam as shown in Equation (2) [13].

(2) $$k=\frac{wt^{3}E}{4L^{3}}$$

where w, L, and t represent the width, the length, and the thickness of the microcantilever, respectively and E represents the Young’s modulus of the beam, which is dictated by its material composition.

When displaced the suspended beam does not follow a linear path but instead bends on a radius. For this reason the effective Young’s modulus characterized by Equation (3) [14] was used in place of E,

(3) $$E\prime =\frac{E}{\left(1-\nu^{2}\right)}$$

where E′ and ν represent the effective Young’s modulus and the Poisson’s ratio of the microcantilever, respectively.

The electrostatic force that acts against the restoring force of the microcantilever is shown in Equation (4) [13],

(4) $$F_{e}=\frac{\varepsilon_{0}A_{de}V^{2}}{2{\left(h_{0}-z\right)}^{2}}$$

where $\varepsilon_{0}$, $A_{de}$, V and $h_{0}$ represent the permittivity of free space, the overlapping area between the suspended beam and the bottom electrode, the applied bias voltage and the initial air cavity height, respectively.

While in operation, the restoring force shown in Equation (1) must be equal to the electrostatic force shown in Equation (4). Should the electrostatic force acting on the suspended beam cause an out of plane displacement in excess of $\frac{h_{0}}{3}$, the beam will collapse causing irreversible damage to the device. The maximum bias voltage that can be applied to the microcantilever without causing the suspended beam to collapse is known as pull-in voltage and is deduced below in Equation (5) [13],

(5) $$V_{PI}=\sqrt{\frac{8}{27}\frac{h_{0}{}^{3}k}{\varepsilon_{0}A_{de}}}$$

Equation (6) [13] characterizes the effective spring constant, which accounts for the material spring constant and the electrostatic force generated from the bias voltage,

(6) $$k_{eff}=\;\frac{wt^{3}E^{\prime }}{4l^{3}}-\frac{\varepsilon_{0}A_{de}V^{2}}{{\left(h_{0}-z\right)}^{3}}$$

where $k_{eff}$ represents the effective spring constant.

Finally, Equation (7) [13] shows the relationship between the effective spring constant and the resonant frequency of the microcantilever,

(7) $$f=\frac{\sqrt{k_{eff}}}{4\pi^{2}\sqrt{0.235m}}$$

where f and m represent the resonant frequency and the equivalent mass of the system, respectively.

### 3.2. Sensitivity Analysis

The sensitivity for mass sensors is defined as a frequency shift from the baseline resonant frequency of the mass sensor per unit mass of analyte, often in the range of picograms (pg), as shown in Equation (8) [15],

(8) $$S=\frac{\Delta f}{m}$$

Referring to Equation (7), it is observed that the effective spring constant $k_{\mathrm{eff}}$ is directly proportional to the resonant frequency of the mass loaded microcantilever structure. For this reason, a change in mass, m, has a greater effect on the resonant frequency if the effective spring constant of the device is increased while under operation. From Equation (6), it is observed that the Young’s modulus and the cross-sectional area of the suspended beam affect the effective spring constant, $k_{eff}$, and subsequently the sensitivity. To optimize and increase the sensitivity of the device two approaches are often considered; geometric optimization and material improvements [16,17,18,19,20,21,22,23,24,25,26,27,28,29,30,31,32,33,34]. Geometric optimization methods that have been explored include implementing multiple suspended beams that converge at the sensing layer and experimenting with non-rectangular beam geometries [10]. In regards to material improvements, the implementation of materials that have a higher Young’s modulus relative to silicon; such as silicon nitride [35] and silicon carbide [36] allow for an increased sensitivity.

In addition to sensitivity, another important parameter to take into consideration is that of the quality factor, which defines the rate of energy dissipation of the device. The quality factor of a microcantilever mass sensor is defined in Equation (9) [37],

(9) $$Q_{C}=b{\left(\frac{L}{t}\right)}^{3}$$

where b represents the damping factor [38]. A system with a higher quality factor dissipates its energy slower than that of a system with a lower quality factor.

The common microcantilever mass sensor’s range of sensitivity is reported beginning at 0.07 Hz/pg [39] with improvement up to 500 kHz/pg [40,41,42].

**Figure 5:**
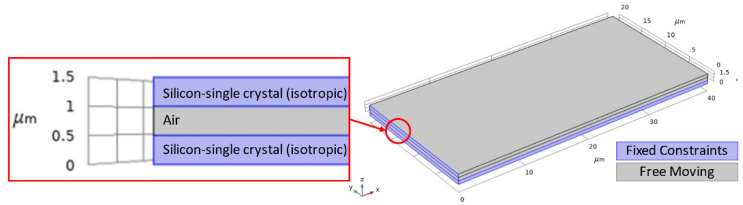
COMSOL Multiphysics^®^ software simulated microcantilever geometry with a length and width of 40 µm and 20 µm, respectively. The top and bottom layers are composed of silicon-single crystal (isotropic) and the middle layer, which forms the cavity is an air domain. The top electrode, air cavity, and bottom electrode each have a thickness of 0.5 µm. Boundaries shown in blue are fixed while the grey boundaries are free to move.

In this work, finite element analysis (FEA) simulations are conducted for the designed microcantilever structure in COMSOL Multiphysics^®^ software (COMSOL Multiphysics^®^ v. 5.4., Stockholm, Sweden) [43] to obtain the microcantilever sensitivity for a target mass change variation between 0.16 and 0.4 pg with a step size of 0.04 pg. Figure 5 shows the geometry of the simulated microcantilever with a length width and thickness of 40 µm, 20 µm, and 0.5 µm, respectively. There was an air cavity of 0.5 µm between the top electrode and bottom electrode both of which were composed of silicon. The left edge of the structure along with the substrate were fixed constraints and the mass was added to the top boundary of the upper block. The user-controlled mesh utilized lesser elements when compared to the physics controlled fine mesh while providing the same results. Thus, the user-controlled mesh was adopted to mesh the model. In this simulation, a fine, tetrahedral mesh was applied to the geometry consisting of 3 domains, 16 boundaries, 28 edges, and 16 vertices resulting in a total of 26,296 domain, 11,744 boundary, and 588 edge mesh elements. The material properties for the COMSOL Multiphysics^®^ software geometry are detailed in Table 1.

**Figure 6:**
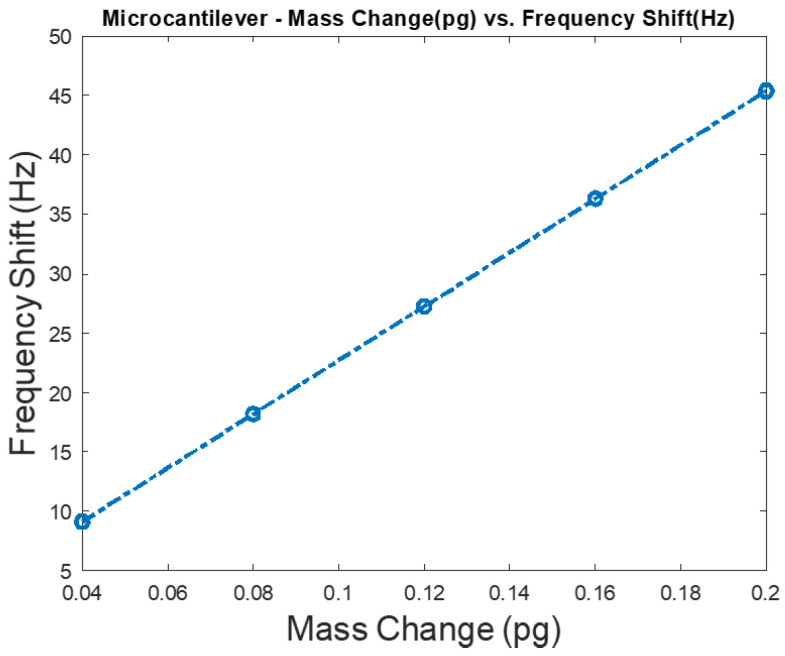
Sensitivity analysis for a silicon microcantilever mass sensor when the target adsorbed mass is increased from 0.16 to 0.4 pg with a step size of 0.04 pg. The investigated sensor has a 40 μm length, a 20 μm width, a 0.5 µm thickness, a cavity height of 0.5 μm, and is made of silicon. A bias of 3 V DC, which is chosen close to the device’s pull-in voltage, is applied to the microcantilever mass sensor.

**Figure 7:**
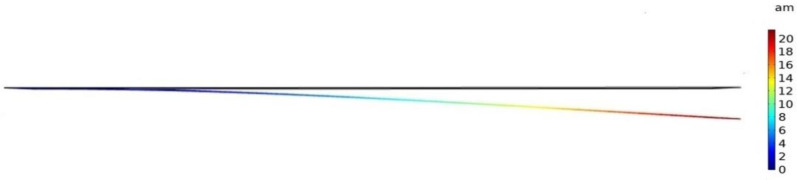
Deformation in a simulated profile for a silicon microcantilever with a width, length, and thickness of 20 µm, 40 µm, and 0.5 µm, respectively. Maximum deflection of about 21 am is obtained in the unclamped free end of the suspended beam, when the microcantilever has a 500 nm cavity height while biased with 3 V DC.

Mass change versus frequency shift is shown in Figure 6 for a microcantilever mass sensor, which includes a flexible beam that is made of silicon with a length, width, and thickness of 40 µm, 20 µm, and 0.5 µm, respectively. In these FEA simulations, the microcantilever is biased with 3 V DC that is chosen close to the device’s pull-in voltage, which results in a maximum deflection of 21 attometers (am) towards the grounded bottom electrode as shown in Figure 7. Solid mechanics and electrostatics effects in the microcantilever structure were investigated through the stationary and eigenfrequency studies when user controlled fine mesh was applied to the structure with tetrahedral components.

### 3.3. Advancements in the Microcantilever Structure

**Figure 8:**
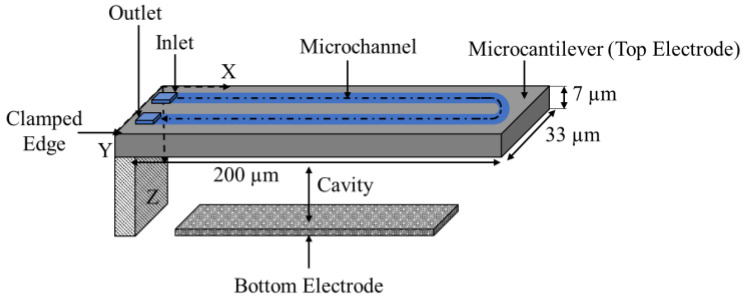
Schematic view of a microchannel microcantilever structure (image not to scale).

The microchannel microcantilever mass sensor was composed of crystal silicon and as the name suggests it includes an embedded channel, which runs through the suspended beam, as shown in Figure 8. The embedded microchannel had a width and height of 8 µm and 3 µm, respectively [44]. The suspended beam structure had a length, width and height of 200 µm, 33 µm, and 7 µm, respectively [44]. The added mass was detected by a shift in resonant frequency of the device upon electrostatic actuation. The air-filled resonator had a resonant frequency of 220.5 kHz, which dropped to 209.6 kHz when injected with water [44].

**Figure 9:**
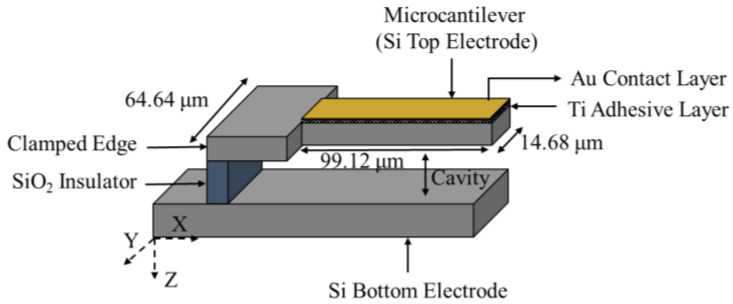
Schematic view of a step-optimized microcantilever structure (image not to scale).

The microcantilever with an enlarged clamped area was etched from an n-type SOI wafer, as shown in Figure 9. The electrodes were separated by a layer of silicon oxide [45]. The device was functionalized by a 0.2 µm thick gold layer adhered to the surface by titanium [45]. The dimensions of the larger, clamped region of the suspended beam were 64.64 µm, 135.46 µm, and 1 µm for width, length, and thickness, respectively [45]. The thinner portion of the highest performing device in this work was 14.68 µm, 99.12 µm, and 1 µm for width, length, and thickness, respectively. The resonant frequency of the unperturbed resonator was reported as 334.75 kHz, which dropped to 334.4 kHz when exposed to an ozone concentration of 180 ppm in a 17250 cm^3^ closed glass chamber [45].

## 4. Microbridge Mass Sensors

### 4.1. Mechanism of Operation

**Figure 10:**
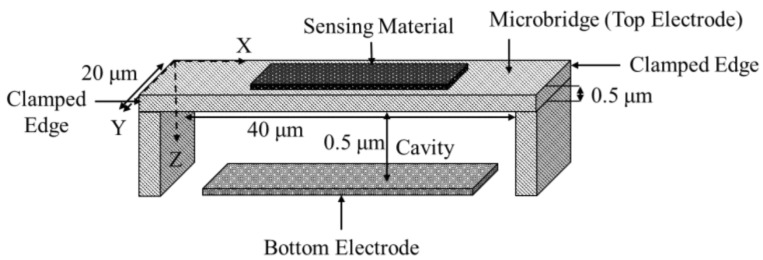
Schematic view of a microbridge structure (image not to scale).

A microbridge mass sensor structure consists of a clamped-clamped beam with a length in the range of hundreds of nanometers to hundreds of micrometers, which is suspended over a bottom electrode [46,47,48]. A cavity is formed between the beam and the bottom electrode, as shown in Figure 10. A microbridge mass sensor can be partially or fully functionalized by a sensing material to detect a target gas [48]. When the sensing material adsorbed the target gas molecules, the mass of the microbridge increased, which resulted in a shift and reduction in the resonant frequency of the sensor. Similar to microcantilever mass sensors, measuring this frequency shift was the main approach in the target gas concentration detection.

This sensor can be actuated based on different techniques including thermal, electrostatic, piezoelectric, and electromagnetic [49]. One of the most commonly used techniques employed in the microbridge structures is that of electrostatic actuation [48,49,50,51]. Similar to microcantilever mass sensors, microbridges are modeled using a mass-spring-damper, as shown in Figure 4, which includes the mass of the structure, the damping factor and the added mass due to the adsorbed analyte [52]. In electrostatic actuation, the bottom electrode is grounded while the microbridge is biased with a DC bias voltage. Similar to microcantilever mass sensors, applied voltage should stay below the pull-in voltage as shown in Equation (5), in order to avoid the device collapsing. The created electrostatic force across the cavity makes the microbridge structure deflect towards the bottom electrode. The maximum displacement of a microbridge with a rectangular cross section can be calculated by Equation (10),

(10) $$\omega_{max}\;=\frac{\rho gL_{b}^{4}}{32E_{b}h^{2}}$$

where ρ, $L_{b}$, g, $E_{b},$ and h represent density, length, gravitational acceleration, Young’s modulus, and thickness of the microbridge, respectively [53].

In a narrow microbridge with small deflections, a differential equation is employed to derive the n^th^ mode of the resonant frequency, as shown in Equation (11) [53],

(11) $$f_{n}=\frac{k_{n}^{2}}{2\pi L_{b}^{2}}\sqrt{\frac{E_{b}I}{\rho A_{b}}}$$

where $k_{n}$, I, and $A_{b}$ represent the microbridge spring constant, the moment of inertia and the cross-sectional area of the microbridge, respectively. $k_{n}$ is given by Equation (12) for frequency modes higher than three, where this value was calculated to be 4.7, 7.8, and 11 for the first, second, and third vibration modes respectively, which are denoted by *n* [53]

(12) $$k_{n}\;\approx \left(n\pi +\frac{\pi }{2}\right)$$

In a microbridge mass sensor, the structure is under a nonlinear stretch due to the applied DC bias voltage, V DC, a result of uniform pressure along the *y*-axis, as shown in Figure 10. Von Karman stretching is employed to derive the equation of motion for a microbridge, as shown in Equation (13) [48,54],

(13) $${E^{\prime }}_{b}I\frac{\partial^{4}\omega }{\partial x^{4}}+\left[\rho bh+\frac{m\left(x\right)}{L_{b}}\right]\frac{\partial^{2}\omega }{\partial t^{2}}=[\frac{{E^{\prime }}_{b}A_{b}}{2L}\int_{0}^{L_{b}}{\left(\frac{\partial \omega }{\partial x}\right)}^{2}dx+F_{e}]\frac{\partial^{2}\omega }{\partial x^{2}}$$

where x, m(x), h, $b,\;\mathrm{and}\;F_{e}$ are position along the microbridge, distributed mass at point *x*, thickness, width, and applied electrostatic force, to the bridge, respectively [54]. $E_{b}^{\prime }$ is the effective Young’s modulus due to the microbridge’s nonlinearity, as previously shown in Equation (3). By applying the boundary conditions and perturbation method into Equation (13), $\beta^{\left(n\right)}$, the ratio of the frequency shift and frequency for the $n^{th}$ vibration mode when mass is deposited at length $L_{b}$ of the microbridge, is derived as shown in Equation (14) [48,50,55],

(14) $$\beta^{\left(n\right)}=\frac{\Delta \omega^{\left(n\right)}}{\omega_{0}^{\left(n\right)}}=-\alpha_{1}\frac{\emptyset_{0}^{\left(n\right)}\left(L_{b}\right)\psi^{\left(n\right)}\left(L_{b}\right)}{2\int_{0}^{1}\emptyset_{0}^{\left(n\right)}\left(x\right)\psi^{\left(n\right)}\left(x\right)dx}$$

where $\alpha_{1}\;$ is the mass ratio that can be calculated using Equation (15). ψ(x)  is the adjoint of $\emptyset_{0}$, where $\emptyset_{0}$ represents the eigenfunction of the unperturbed microbridge

(15) $$\alpha_{1}=\frac{m\left(x\right)}{\rho bhL_{b}}.$$

### 4.2. Sensitivity Analysis

Similar to microcantilever mass sensors, sensitivity is defined as the frequency shift per unit mass, previously shown in Equation (8), $S=\frac{\Delta f}{m}$, where Δf and m represent the frequency shift and mass, respectively. In order to design a high-sensitivity microbridge mass sensor, the length of the structure can be decreased in addition to measuring the device while operating at higher frequency modes [48]. The study on the higher modes illustrates that these modes contribute differently in achieving higher sensitivity. This phenomenon depends on the position of the added mass on a microbridge due to the sensing material and consequently the adsorbed target gas molecules [48]. For instance, measuring the first mode shows the highest sensitivity when the sensing material is deposited on the middle of the bridge. However, the symmetric points, 0.3 and 0.7 of the total length of the microbridge, and the points 0.2 and 0.8, contribute to the maximum achieved sensitivity for the second and third modes of the frequency, respectively [50].

One of the important parameters in designing microbridge mass sensors is the quality factor, which illustrates the ratio of the stored energy to the dissipated energy per vibration cycle [56]. It can be calculated by damping factor b, as shown in Equation (16), where k is the microbridge spring constant [57],

(16) $$Q_{b}=\frac{\sqrt{k\;m}}{b}$$

A higher quality factor illustrates that more energy remains in the system than the used to dampen the structure, therefore, based on Equation (16), a stiffer bridge structure can be correlated to a higher device performance.

The common microbridge mass sensor’s range of sensitivity is reported between a few Hz/pg with an improvement to tens of Hz/zg, when it is functionalized with carbon nanotubes (CNTs) [50].

In this work, FEA simulations were conducted using COMSOL Multiphysics^®^ software for a microbridge mass sensor to investigate the sensitivity when the adsorbed target gas was changed between 0.16 and 0.4 pg with a step size of 0.04 pg.

**Figure 11:**
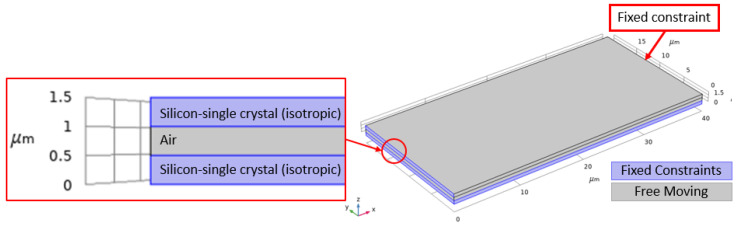
COMSOL Multiphysics^®^ software simulated microbridge geometry with a length and width of 40 µm and 20 µm, respectively. The top and bottom layers are composed of silicon-single crystal (isotropic) and the middle layer, which forms the cavity is an air domain. The top electrode, air cavity and bottom electrode each have a thickness of 0.5 µm. Boundaries shown in blue are fixed while the grey boundaries are free to move.

Figure 11 shows the geometry of the simulated microbridge with a length width and thickness of 40 µm, 20 µm, and 0.5 µm, respectively. There was an air cavity of 0.5 µm between the top electrode and bottom electrode both of which were composed of silicon. The left and right edges of the structure along with the substrate were fixed constraints and the mass was added to the top boundary of the upper block. The user-controlled mesh utilizes lesser elements when compared to the physics controlled fine mesh while providing the same results. Thus, the user-controlled mesh was adopted to mesh the model. In this simulation, a fine, tetrahedral mesh was applied to the geometry consisting of 3 domains, 16 boundaries, 28 edges, and 16 vertices resulting in a total of 26,296 domain, 11,744 boundary, and 588 edge mesh elements. The material properties for the COMSOL Multiphysics^®^ software geometry are detailed in Table 1.

**Figure 12:**
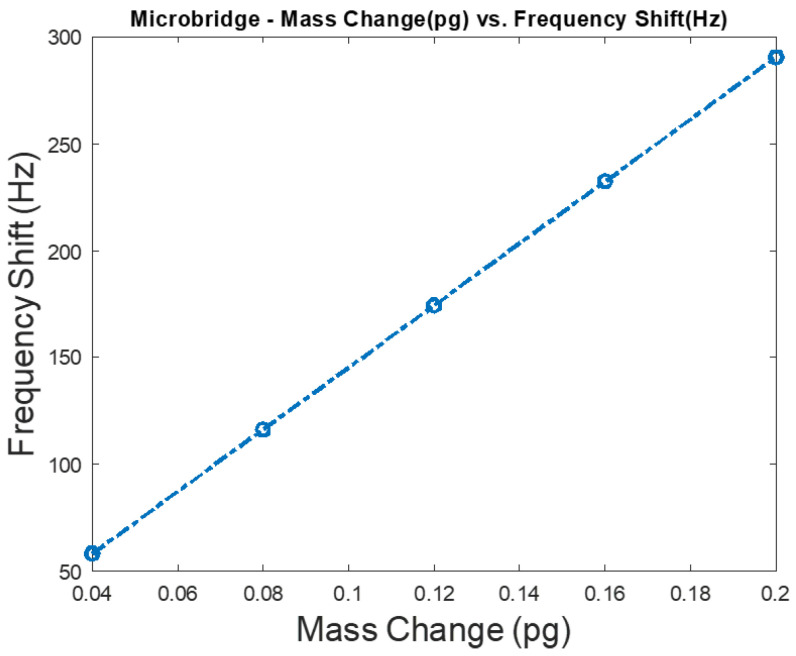
Sensitivity analysis for a silicon bridge mass sensor when the target adsorbed mass is increased from 0.16 to 0.4 pg. The investigated sensor has 40 μm length and 20 μm width when the thickness and cavity are 0.5 μm. A bias of 20 V DC, which is chosen close to the device’s pull-in voltage, is applied to the microbridge mass sensor.

**Figure 13:**
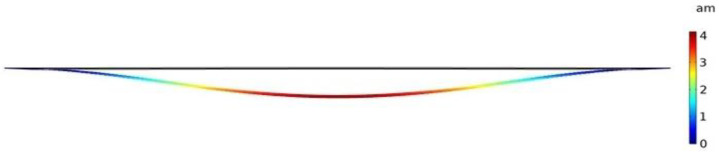
Deformation in a simulated profile for a silicon microbridge when width, length, and thickness are 20 µm, 40 µm, and 0.5 µm, respectively. Maximum deflection of about 4 am is obtained in the middle of the structure, when the microbridge has a 500 nm cavity height while biased with 20 V DC_._

Frequency shift versus mass change is shown in Figure 12 for a silicon microbridge mass sensor with a 40 μm length and a 20 μm width, when the thickness and the cavity height were 0.5 μm each. In these FEA simulations, the microbridge was clamped at each end and the sensor was biased with 20 V DC that was chosen close to the device’s pull-in voltage, which resulted in a deflection towards the grounded bottom electrode. Solid mechanics and electrostatics effects in the microbridge were investigated through the stationary and eigenfrequency studies when user controlled fine mesh is applied to the structure with tetrahedral components. COMSOL Multiphysics^®^ software simulations show that the maximum deflection occurs in the middle of the microbridge, while the edges are clamped, as shown in Figure 13.

### 4.3. Advancements in the Microbridge Structure

**Figure 14:**
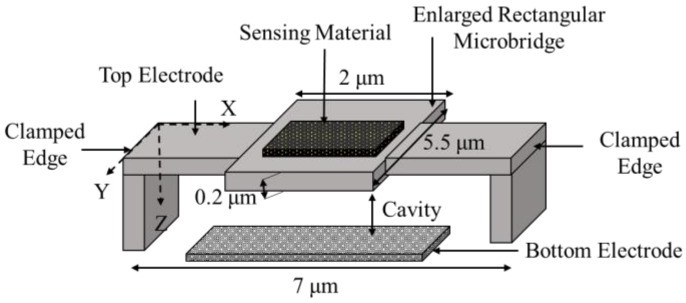
Schematic view of an enlarged rectangular microbridge structure (image not to scale).

The microbridge mass sensor is developed to a polysilicon microbridge with an enlarged rectangular area at the center, which is fabricated by employing a sacrificial technique, as shown in Figure 14. The enlarged center, which was used as a mass detection area, had 5.5 µm length, 2 µm width, and 0.2 µm thickness, when the total length of the microbridge was 7 µm [46]. This structure was locally coated for mass detection applications. In this microbridge mass sensor, the added mass was measured by the shift in either bending or torsion resonant frequencies depending on the length and thickness ratio of the structure. Reported resonant frequencies were 5 MHz and 12 MHz for torsion and bending modes, respectively.

**Figure 15:**
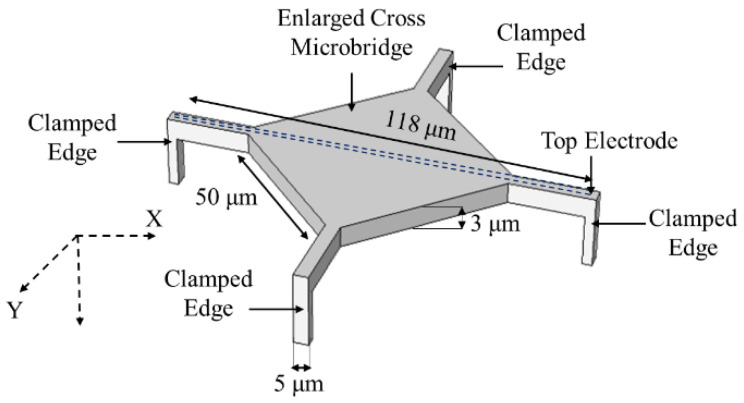
Schematic view of an enlarged cross microbridge structure (image not to scale).

This design is further developed to a silicon cross bridge structure with 78 MHz resonant frequency and fabricated using wafer bonding technique, as shown in Figure 15. In this design the total length and width of the bridge, the square dimension and thickness were 118 µm, 5 µm, 50 µm, and 3 µm, respectively [58]. This structure was used for fluids detections when the liquid went through integrated microchannels in bridge and the sensor operates in a dry environment. This structure benefits from enhanced quality factor, while measuring fluid mass changes [58].

## 5. Clamped Membrane Mass Sensors

### 5.1. Mechanism of Operation

**Figure 16:**
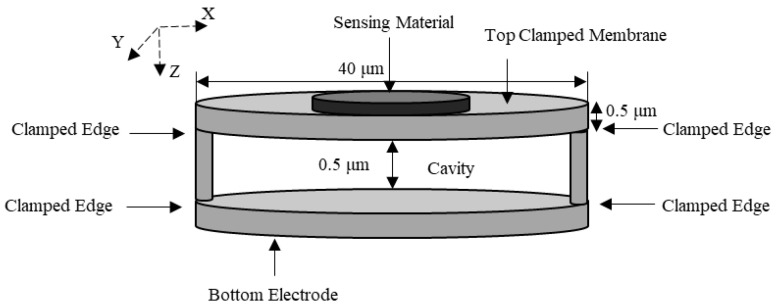
Schematic view of a capacitive clamped membrane structure (*x*–*z* axis perspective) (image not to scale).

**Figure 17:**
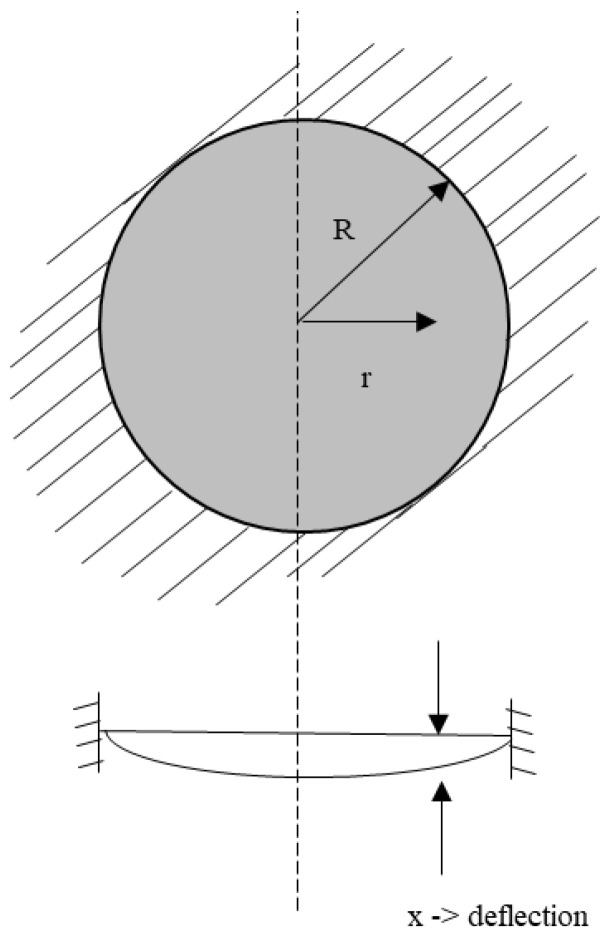
Top cross-sectional view of a capacitive clamped membrane structure (*x*–*y* axis perspective).

A capacitive clamped membrane (CCM) sensor is commonly composed of a flexible membrane of either silicon or polysilicon, which is fully clamped on all sides with a range of dimensions in the order of tens of micrometers to achieve resonant frequencies in the MHz range [59]. This flexible membrane is suspended over a bottom electrode forming an air cavity in between [59,60,61,62]. The mechanics of a CCM can be explained through plate theory [60,61,62,63,64,65]. CCM sensors are employed as gas sensors, functionalized by a sensing material deposited on the flexible membrane. The sensing material adsorbs target gas molecules, increasing the overall mass of the top membrane. Figure 16 illustrates a cross-sectional view of a circular CCM sensor while Figure 17 shows the top view of the same device from the *x*–*y* axis perspective.

Similar to microcantilever and microbridge mass sensors, capacitive clamped membranes are also modeled using a mass-spring-damper, as shown in Figure 4, which includes the mass of the structure, the damping factor and the added mass due to the adsorbed analyte [52]. These devices are electrostatically actuated, which causes an attractive electrostatic force between the flexible membrane and the fixed bottom electrode, pulling the former towards the latter known as the pull-in effect. Similar to the microcantilever and the microbridge, this pull-in effect is balanced out by the restoring force of the membrane. The volatile organic compound adsorbed by the sensing material increases the mass of the top clamped membrane, hence creating an additional force in the negative z-direction. The added mass disrupts this intricate balance between the electrostatic and restoring forces, causing the membrane to deflect towards the bottom electrode, thereby reducing the height of the air cavity. The reduction in air cavity height causes a measurable increase in the CCM capacitance shown in the device resonant frequency. Therefore, a relationship can be established between loaded mass and resonant frequency of the structure allowing the CCM structure to act as a mass sensor.

If the applied bias voltage is increased beyond a certain point, it adversely affects the structural integrity of the membrane, causing it to fully collapse and make contact with the bottom electrode, ultimately damaging the device. Therefore, the CCM sensor is operated below this maximum voltage potential known as the pull-in voltage (*V_PI_*).

The attractive force between the flexible membrane and the fixed electrode is given by,

(17) $$F_{e}=\;\frac{-d}{dz}\left(\frac{CV^{2}}{2}\right)=\frac{\varepsilon_{0}A_{m}V^{2}}{2{\left(h_{0}-z\right)}^{2}}$$

where $h_{0}$, $A_{m}$, V, and z refer to the initial unbiased cavity height, the flexible membrane’s area, the applied DC bias and the out-of-plane displacement of the membrane, respectively [66].

The restoring mechanical force is given by the Hooke’s Law, previously shown in Equation (1), where F and k represent the applied mechanical force in newtons and the spring constant, respectively.

(18) $$\;k=\frac{16\;\pi \;E_{m}\;t_{m}^{3}}{3\;\left(1-\upsilon_{m}^{2}\right)\;r_{m}^{2}}-\;\frac{\varepsilon_{0}\;A_{m}\;V^{2}\;}{d_{eff}^{3}}+\;4\pi \sigma_{m}t_{m}$$

In Equation (18), $E_{m}$, $t_{m}$, $r_{m}$, $\upsilon_{m}$, $\sigma_{m},$ and $d_{eff}$ refer to the Young’s modulus, thickness, radius, Poisson’s ratio, residual stress of the clamped membrane, and the effective cavity height of the device, respectively [59,67]. The first term in Equation (18) is due to the geometry shown in Figure 16, whereas the second and third terms are the spring softening effect due to the DC potential applied across the membranes and the residual stress of the top clamped membrane.

The pull-in voltage occurs at 1/3 of the cavity height, and it is derived by combining Equation (17) and Equation (1), which is then derived by dz and equated to 0, as shown by Equation (5) [68]. To prevent this collapse, and consequent shorting of the now parallel-plate capacitor, an insulator may be introduced above the bottom electrode [62,69].

This addition changes the initial cavity height to the effective cavity height,

(19) $$h_{eff}=\;\frac{t_{r}}{\varepsilon_{r}}+\;h_{0}$$

where $t_{r}$ and $\varepsilon_{r}$ are the insulation layer’s thickness and the insulator’s relative dielectric permittivity, respectively. Hence, now the capacitance can be calculated by using the effective gap height,

(20) $$C\left(z\right)=\frac{A_{m}\varepsilon_{0}}{h_{eff}-z}$$

To find the membrane’s resonant frequency [67,70], Equation (21) is used:

(21) $$\omega_{r}=2\pi f\;=\;\sqrt{\frac{k}{m_{m}}}$$

where $\omega_{r}$ and $m_{m}$ refer to the angular resonant frequency and the effective mass of the membrane, respectively.

As a mass sensor, there is a sensing layer on top of the suspended membrane. Therefore, the spring constant k in Equation (18) is modified to $k_{bi}$ in Equation (22) [67]. $k_{bi}$ accounts for the addition of a sensing layer and its consequent residual stresses

(22) $$k_{bi}=\frac{64\pi D_{eff}}{\;r_{m}^{2}}-\;\frac{\varepsilon_{0}\;A_{m}\;V^{2}\;}{d_{eff}^{3}}+\;4\pi \left(\sigma_{m}t_{m}+\sigma_{s}t_{s}\right)$$

where $\sigma_{s}$, $t_{s}$, and $D_{eff}$ refer to the sensing layer residual stress, the sensing layer thickness and the effective flexural rigidity, respectively.

### 5.2. Sensitivity Analysis

Similar to the microcantilever mass sensors and the bridge mass sensors, sensitivity is defined as frequency shift per unit mass of adsorbed analyte by the sensing membrane as previously shown in Equation (8), $S=\frac{\Delta f}{m}$, where Δf and m represent the frequency shift and the mass, respectively [67].

The mass sensitivity per unit area is defined as [71,72],

(23) $$S_{m}=\;-2\frac{m}{A_{m}f_{0}}=-\frac{2\rho t}{f_{0}}$$

where $f_{0}$ refers to initial resonant frequency.

Whereas the mass resolution, which is limited by the noise level, for the capacitive clamped membrane is defined as,

(24) $$\frac{\Delta m}{A_{m}}=-\frac{2\rho t}{f}f_{noise}$$

where $f_{noise}$ refers to the frequency noise [73].

A high-quality factor contributes to a decrease in the frequency noise. The quality factor in the frequency domain is defined as,

(25) $$Q_{fd}=\frac{f}{f_{bandwidth}}$$

where $f_{bandwidth}$ refers to the frequency bandwidth.

In the physical domain the quality factor is defined as,

(26) $$Q_{pd}=\frac{E_{s}}{W_{c}}*\;2\pi$$

where $E_{s}$ refers to the energy stored in the capacitive clamped membrane as a resonator, and $W_{c}$ is the energy lost in a singly resonating cycle τ,

(27) $$\tau =\frac{1}{f}.$$

For a capacitive clamped membrane, the quality factor is majorly affected by air loss followed by the support loss, which are defined as,

(28) $$Q_{air}=\frac{2\pi \rho t}{Z_{a}}f$$

where $Z_{a}$ refers to the acoustical impedance of air [73], and

(29) $$Q_{support}=0.638{\left(\frac{L}{t}\right)}^{3}$$

at the first mode of resonance, respectively.

The common capacitive clamped mass sensor’s range of sensitivity when configured in arrays and having smaller cavities and thinner membranes is reported between 48.8 Hz/zg and 130 Hz/zg when functionalized by poly{methyl[3-(2-hydroxyl, 4,6-bistrifluoromethyl)phenyl]propylsiloxane} (DKAP) and polyisobutylene (PIB), respectively [72,74].

In this work, FEA simulations are conducted for the capacitive clamped membrane in COMSOL Multiphysics^®^ software to investigate the change of sensitivity for different added analyte masses, which ranged from 0.25 to 0.63 pg with a step size of 0.063 pg.

**Figure 18:**
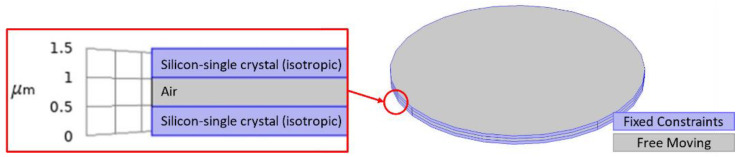
COMSOL Multiphysics^®^ software simulated clamped membrane geometry with a 40 µm diameter. The top and bottom layers are composed of silicon-single crystal (isotropic) and the middle layer is an air cavity. The thickness of the top and bottom membranes and the air cavity height are 0.5 µm each. Boundaries shown in blue are fixed while the grey boundaries are free to move.

Figure 18 shows the geometry of the simulated clamped membrane with a 40 µm diameter when the thickness of the top and bottom membrane was 0.5 µm. There was an air cavity of 0.5 µm between the top and bottom membranes, both of which were composed of silicon. The edges of the silicon top membrane along with the bottom membrane were fixed constraints and the mass was added to the top boundary of the top membrane. The user-controlled mesh utilized lesser elements when compared to the physics controlled fine mesh while providing the same results. Thus, the user-controlled mesh was adopted to mesh the model. In this simulation, a fine, tetrahedral mesh was applied to the geometry consisting of 3 domains, 16 boundaries, 28 edges, and 16 vertices resulting in a total of 50,847 domain elements, 21,120 boundary elements, and 620 edge elements. The material properties for the COMSOL Multiphysics^®^ software geometry are detailed in Table 1.

**Figure 19:**
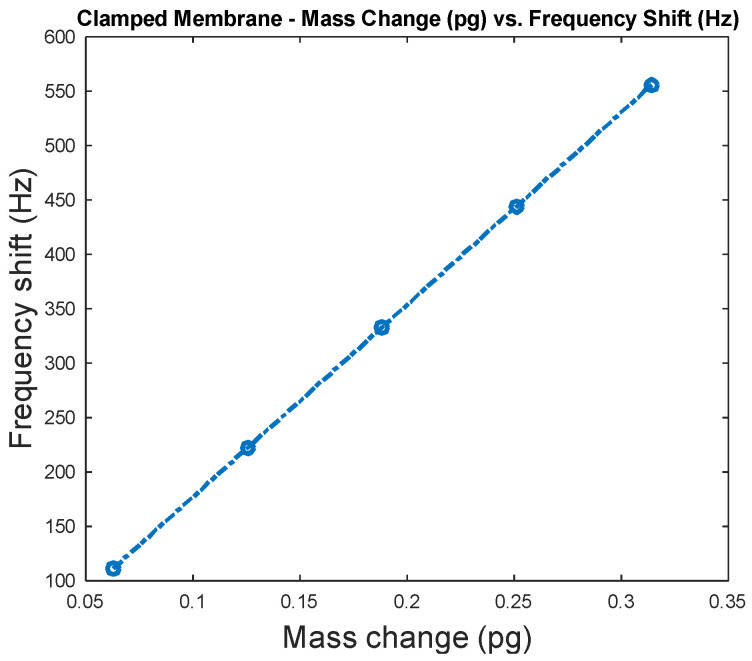
Sensitivity analysis for a silicon capacitive clamped membrane sensor when the target adsorbed mass is increased from 0.25 to 0.63 pg with a step size of 0.063 pg. The investigated sensor has a 40 μm diameter, when the thickness of the top and bottom membranes and the cavity height are 0.5 μm each. A bias of 20 V DC, which is chosen close to the device’s pull-in voltage, is applied to the capacitive clamped membrane mass sensor.

**Figure 20:**
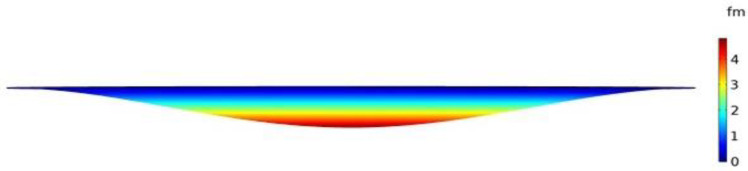
Deformation in a simulated profile for a silicon capacitive clamped membrane with a 20 µm radius and 500 nm membrane thickness. Maximum deflection of about 4 femtometers (fm) is obtained in the middle of the structure, when the capacitive clamped membrane has a 500 nm cavity height while biased with 20 V DC.

Frequency shift versus mass change is shown in Figure 19 for a silicon capacitive clamped membrane mass sensor with 40 μm diameter when the thickness of the top and bottom membrane and the cavity height were 0.5 μm. In these FEA simulations, the capacitive membrane was fully clamped, and the sensor was biased with 20 V DC, which is chosen close to the device’s pull-in voltage, which results in a deflection towards the grounded bottom electrode. Solid mechanics and electrostatics effects in the capacitive clamped membrane are investigated through the stationary and eigenfrequency studies when user controlled fine mesh is applied to the structure with tetrahedral components. COMSOL Multiphysics^®^ software simulations show that the maximum deflection occurs in the middle of the clamped membrane, while it is fully clamped, as shown in Figure 20.

### 5.3. Advancements in the Capacitive Clamped Membrane Structure

**Figure 21:**
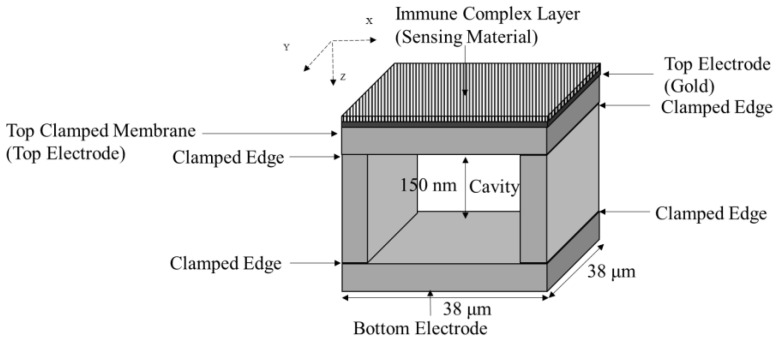
Schematic view of a square capacitive clamped membrane structure (image not to scale).

The CCM mass sensor is developed into a square CCM [75], which is fabricated by a wafer bonding technique, as shown in Figure 21.

This square CCM has a highly doped and oxidized substrate and a 1 μm thickness square silicon monocrystal directly bonded to form a 38 µm × 38 μm cell. A cavity of 150 nm is formed in the cell and the structure is coated with 300 nm gold electrodes. The square CCM was functionalized by an immune complex layer of bovine leukemia virus antigen gp51 (BLV gp51) [75], as shown in Figure 21. The nominal frequency of the fabricated square CCM was 7 MHz.

**Figure 22:**
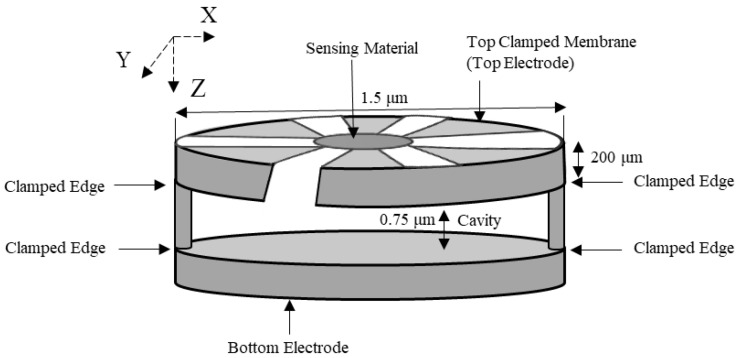
Schematic view of a wheel capacitive clamped membrane structure (image not to scale).

The CCM mass sensor is further developed into a wheel shaped polysilicon diaphragm microresonator (wheel CCM), with an external opening on the side and it is used for carbon dioxide detection, as shown in Figure 22.

This wheel CCM [76] is fabricated by employing a sacrificial technique, PolyMUMPS [77]. The mass detection area is at the center of the wheel shaped structure. The wheel CCM has a thickness, diameter, and achieved cavity height of 200 μm, 1.5 μm, and 0.75 μm, respectively. Any added mass like the conventional CCM was detected through a decrease in the resonant frequency of the wheel CCM. The nominal resonant frequency of the fabricated wheel CCM was 1.15 MHz with a quality factor of 8 at atmospheric pressure and 300 at an ambient pressure of 133.32 Pa [76]. As the PEI coating was applied to the wheel CCM, the structure’s resonant frequency decreased to 1.139 MHz.

## 6. Discussion

Microcantilever mass sensors are often employed due to their low-cost, high sensitivity, low power consumption, fast response, and fabrication simplicity [10,78,79,80,81,82]. Additionally, a high surface to volume ratio offered by microcantilever mass sensors makes it possible to identify low level mass detection of biological and chemical agents as compared to classical mass detection methods [83,84,85], which often require skilled personnel to implement [10].

Amongst the many advantages offered by a microcantilever mass sensor, there exist some notable disadvantages such as undesirable deflections, commonly referred to as parasitic deflections [4,38]. These parasitic deflections are normally caused by the physical adsorption of non-specific molecules on the surface of the unclamped free end of the microcantilever [4,38]. The added mass of the non-specific molecules results in a shift of the baseline resonant frequency of the device. To track the change in the baseline resonant frequency as a result of these environmental interactions, often a reference microcantilever is utilized without a sensing layer [4,38].

While microcantilever mass sensors have proven useful in detecting static particle masses as low as 7 zg when placed in a vacuum, the performance of these devices is significantly reduced when placed in a solution [15]. A conventional microcantilever may achieve quality factors up to 15,000 [15], which are significantly reduced when submersed in a liquid due to viscous drag with quality factors of less than 200 [15,16]. This reduction in the quality factor severely limits this device’s usefulness in biomedical applications.

The microchannel microcantilever structure, shown in Figure 8, is introduced to maintain a high quality factor regardless of the sensing medium, a microfluidic channel can be incorporated into the suspended beam making the sensor employable for both liquid and gas sensing applications. The microchannel circulates the fluid through the suspended beam in a closed loop with access ports at the clamped side of the resonator [15]. The interior walls of the channel are then functionalized, providing a higher effective surface to volume ratio than that of a conventional microcantilever. For transient particle measurements, a sensing layer coated on the interior walls is not necessary for some applications as the flow of a single point mass through the device translates to a shift in resonant frequency [15]. The microfluidic channel microcantilever operates in vacuum, hence, maintaining a high-quality factor regardless of the viscosity of the analyte carrying medium. In this case, gas sensing is potentially achieved by pumping the gas analyte through the microchannel dry or be introduced in combination with a carrier liquid by use of a bubbler should label-free detection be unnecessary for the application.

With regards to the microcantilever geometric shape and profile improvements a microcantilever with a suspended beam thickness ratio; $\frac{h_{1}}{h_{0}}=0.05$ reported a sensitivity increase of 50.9% when compared to a suspended beam thickness ratio of $\frac{h_{1}}{h_{0}}=1$ where, $h_{0}$ and $h_{1}$ represent the thickness of the beam at the clamped end and free end, respectively [86]. Additionally, a triangular shaped suspended beam resulted in two-fold increase in resonant frequency with respect to the traditional rectangular shaped beam of equal functional surface area. To promote analyte adsorption at the tip of the suspended beam, an optimized stepped suspended beam with triangular clamped region was simulated; revealing a sensitivity increase 3.16 times that of a rectangular device of a similar surface area [86]. Devices similar resembling the work of Lui et al. have been fabricated and tested for the detection of ozone [45]. A suspended beam with a functionalized tip as shown in Figure 9 proved to be effective at measure nanogram ozone concentrations of 0.6 ppm/Hz [45].

Microbridge structures are employed for mass detection in gas and liquid phases [46,50,87]. This structure benefits from higher quality factor and operating frequency, in the order of GHz when higher modes are measured, as compared to microcantilevers. These properties are due to the bridge’s higher stiffness, with dimensions similar to microcantilevers, which consequently contributes to a higher mass sensitivity [46,50]. Similar to microcantilever mass sensors, a non-desirable shift in the resonant frequency can occur that can be mitigated through the use of a reference bridge sensor without a sensing material. Furthermore, the microbridge structure shares the same drawback of a lower quality factor when submersed in liquid, which limits the structure in biomedical applications.

The enlarged rectangular microbridge structure, as shown in Figure 14, is introduced as an advancement to a normal microbridge structure. This structure device benefits from a larger detection area while its mass is decreased for enhanced mass sensitivity properties. In addition, monitoring mass deposition is possible in two modes, both bending and torsion modes, due to the symmetricity of the structure. The microbridge with enlarged rectangular detection area also provides a better sensitivity in its bending mode compared to the torsion mode in locating the adsorbed mass applications [46].

In the enlarged cross microbridge structure, as shown in Figure 15, the target gas or liquid flows through the integrated microchannels while the sensor operates in a dry environment promoting a higher quality factor [58]. Similar to the microchannel microcantilever structure, shown in Figure 8, this structure can achieve gas sensing by pumping the gas analyte through the microchannel or with a carrier liquid by use of a bubbler thereby showing potential for implementation in biomedical applications.

Capacitive clamped membranes offer high electromechanical coupling in comparison with other sensors such as piezoelectric structures used for mass sensing [88,89]. The few drawbacks of the capacitive clamped membranes are poor selectivity and baseline drift, which is also the case with microcantilever and microbridge structures. The issue of poor selectivity is addressed by fabricating CCM sensors in arrays with a variety of sensing materials [74,90,91]. Similar to microcantilever and microbridge sensors, baseline drift is addressed by using a reference resonator fabricated without a sensing layer. Furthermore, capacitive clamped membranes offer a higher bandwidth and low concentration level detection of volatile organic compounds [69,72,92,93,94,95]. They are batch produced by using advanced fabrication techniques in microelectromechanical systems such as sacrificial or bonding techniques [67,96,97,98,99,100,101,102,103].

The square CCM, as shown in Figure 21, is used as an immunosensor for bovine leukemia virus. This sealed cavity CCM structure has lesser damping than both microcantilever and microbridge structures, which results in higher resonant frequencies and a higher quality factor [75]. The square CCM structure benefits from the gold electrodes, which provide better electrical coupling with the BLV gp51 antigen as the functionalized sensing layer. Modifying the membrane of the CCM by BLV gp51 through covalent bonds results in improved selectivity towards the antibodies of the bovine leukemia virus [75].

**Figure 23:**
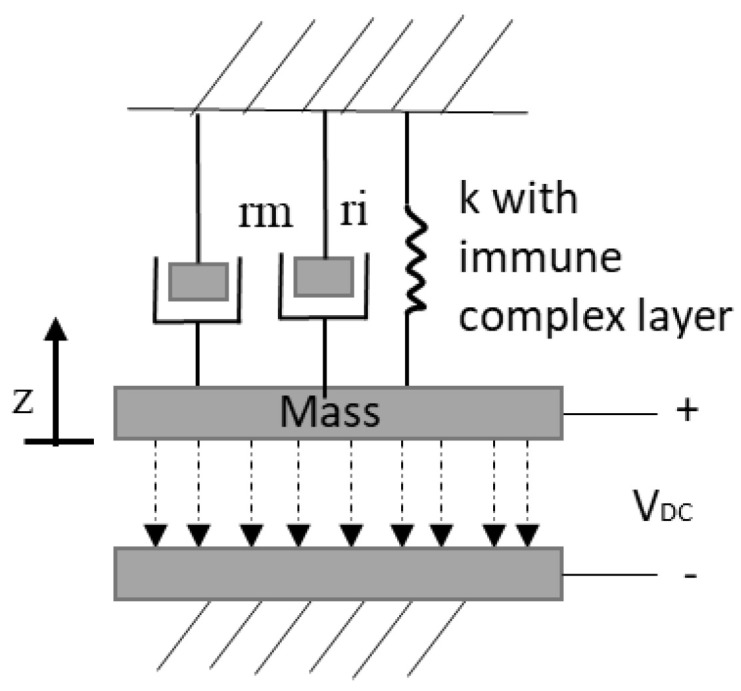
Circular capacitive micromachined ultrasonic transducer (CMUT) bilayer model.

Furthermore, the modification of the top membrane in this structure forces an increase in the resonant frequency of the device. This is in conflict with the mass-spring model, shown in Figure 4, however it can be attributed to an increased stiffness in the spring constant of the membrane due to steps required in the modification of the membrane such as drying, rinsing, and crosslinking, which is required for the reversibility of the immunosensor. This leads to an overall increase in the spring constant where the effect of increasing the mass is traded off with the increase in the stiffness and stress in the structure. Furthermore, the antibodies combining with the BLV gp51 antigen, prompts the added mass to inherit the mass density and elasticity properties of the bovine serum albumine, intrinsically modifying the membrane properties [75]. A possible solution to this would be to employ the bilayer circular capacitive micromachined ultrasonic transducer (CMUT) model [67], as shown in Figure 23, however, tailored specifically towards square membrane CCMs.

The wheel CCM, as shown in Figure 22, was used as a gas sensor for low-level mass detection of carbon dioxide. An external opening in the clamped membrane of this structure is provided to reduce damping due to the air flow passing through the plates of the sensor [76]. Due to the large openings in the wheel CCM, the overall mass of the sensor was reduced, which led to a higher resonant frequency for the structure, as per Equation (18) and Equation (21). However, this also led to a lower quality factor for the structure, which can be traded off by an array configuration of the wheel CCMs and averaging the measurements. This structure is coated with linear polyethylenimine (PEI) solution, which is used for carbon dioxide detection due to its high selectivity, low sensitivity to humidity, and linear reversibility [76].

Sensitivity and applications of different geometry-based mass sensors such as the microcantilever, microbridge, and capacitive clamped membrane are shown in Table 2.

**Figure 24:**
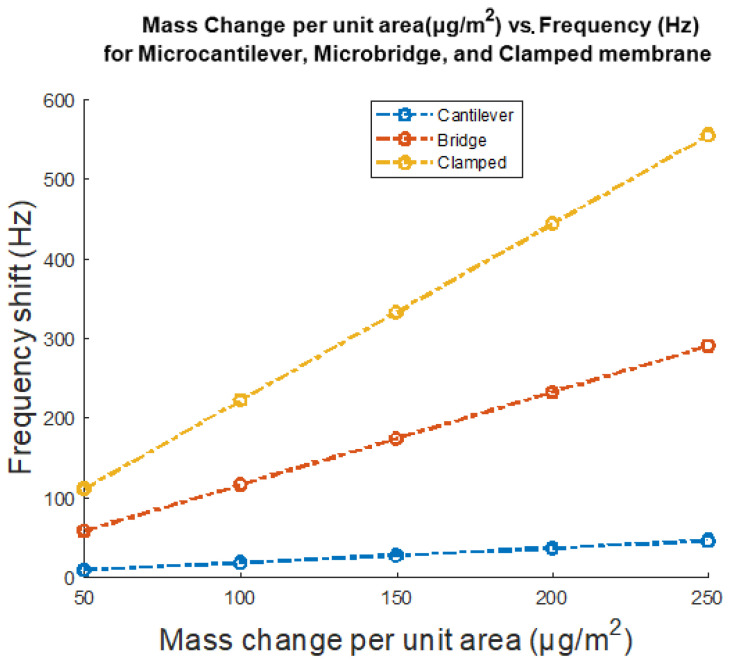
Simulation results are shown for change in frequency (Hz) in response to change in loaded mass per unit area (µg/m^2^) for the three different investigated geometries of the mass sensors, microcantilever, microbridge, and clamped membrane.

Figure 24 shows the simulation results for change in frequency (Hz) in response to change in loaded mass per unit area (µg/m^2^) for the three different investigated geometries of the mass sensors, microcantilever, microbridge, and clamped membrane.

## 7. Conclusions

This paper investigates and categorically classifies the different capacitive based mass sensors according to their different geometries and mechanisms of operation. It gives a thorough understanding of the operational principle of each mass sensor, followed by sensitivity analysis for each mass sensor.

This paper did an investigative analysis of three different geometries of the micromachined mass sensors, microcantilever, microbridge, and clamped membrane. A silicon microcantilever mass sensor and a silicon microbridge mass sensor were simulated where the target adsorbed mass was increased from 0.16 to 0.4 pg with a step size of 0.04 pg. The investigated microcantilever and microbridge mass sensors had a 40 μm length, a 20 μm width, a 0.5 µm thickness, and a cavity height of 0.5 μm each, respectively. The maximum deflection of 21 am was obtained in the unclamped free end of the suspended beam of the microcantilever while it was biased with 3 V DC. Maximum deflection of 4 am was obtained in the middle of the microbridge structure while it was biased with 20 V DC.

A silicon capacitive clamped membrane sensor was simulated where the target adsorbed mass was increased from 0.25 to 0.63 pg with a step size of 0.063 pg. The investigated clamped membrane mass sensor had a 40 μm diameter, when the thickness of the top and bottom membranes and the cavity height were 0.5 μm each. Maximum deflection of 4 fm was obtained in the middle of the clamped membrane structure while it was biased with 20 V DC. The bias voltages were chosen close to each devices’ pull-in voltages.

Figure 24 shows the frequency (Hz) in response to change in loaded mass per unit area (µg/m^2^) for the three different investigated geometries of mass sensors, microcantilever, microbridge, and clamped membrane, wherein it could be seen that the clamped membrane shows the highest frequency shift per mass change per unit area. This could be correlated to the difference of the geometry of the structures and their respective clamping conditions. Furthermore, it is noted that the difference in the material composition of the clamped membrane influenced the sensitivity of the device due to a different Young’s modulus. The difference in the slopes of sensitivity for each mass sensor could be attributed to the varying degrees of clamping between the sensor geometries. As clamping increased, the resonant frequency increased for similar electrostatically actuated structures. Moreover, this investigative review paper discussed the different advancements related to each simulated geometry and gave insight to the advantages and disadvantages associated with each them. Essentially, this paper provided a structured foundation for the common capacitive based micromachined mass sensors and a critical discussion to build the groundwork for any related future work as per need based application.

## Figures and Tables

**Table 1:** Material properties for COMSOL Multiphysics^®^ software simulations for microcantilever, microbridge, and clamped membrane geometries.

Material	Relative Permittivity	Density (kg∙m^−3^)	Young’s Modulus (Pa)	Poisson’s Ratio	Thermal Expansion Coefficient (K^−1^)	Heat Capacity at Constant Pressure (J∙kg^−1^∙K^−1^)	Thermal Conductivity (W∙m^−1^∙K^−1^)
Silicon	11.7	2329	170e9	0.28	2.6e−6	700	130
Air	1	1.225	0	0	-	-	-

**Table 2:** Reported sensitivities and applications for different geometry-based mass sensors.

Sensor	Sensitivity	Applications
Rectangular Microcantilever	32.5 Hz/pg [39]	Carbon Dioxide Detection [104]
	Simulated Sensitivity 226.96 Hz/pg	
Microchannel Microcantilever	3.1–3.6 Hz/pg [105]	Biosensing (E. Coli And B. Subtilis) [105]
Step-Optimized Microcantilever	1.7 Hz/pg, for 1mg of Ozone Concentration [45]	Ozone Detection [45]
Rectangular Microbridge	0.21 Hz/pg [106]	Greenhouse Detection [107]
	Simulated Sensitivity 1.45 kHz/pg	
Microbridge With Enlarged Rectangular Detection Area	femtogram to attogram Range of Detection [46]	Thiol Detection [46]
Cross Microbridge With Enlarged Square Detection Area	0.3 MHz/g/cm^−3^ [27]	Ethanol Detection [27]
Circular Capacitive Clamped Membrane	250 Hz/pg, Mass Resolution of fg [75]	Immunosensing Of Bovine Leukemia Virus [75]
	Simulated Sensitivity in this work: 1.77 kHz/pg	
Square Capacitive Clamped Membrane	488 Hz/pg, Mass Resolution of ag [75].	Immunosensing Of Bovine Leukemia Virus [75].
Wheel Capacitive Clamped Membrane	64.6 kHz /g/cm^−3^ [76]	Carbon Dioxide Detection [76]

## Data Availability

Not applicable.
